# Febrile Periodicity, as Influenced by the Sulphate of Quinine

**Published:** 1845-06

**Authors:** Thos. D. Mitchell

**Affiliations:** Prof. Mat. Med. and Therap., in Transylvania University


*Febrile Periodicity, as influenced by the Sulphate of Quinine.* By Thos. D. Mitchell, M. D., Prof. Mat. Med. and Therap., in Transylvania University.—But a few years ago, the therapeutical application of this invaluable salt of Peruvian bark, was exceedingly limited; and with some, even at this day, it is held to be competent, as a remedy, only to true intermitting fever, or ague and fever.

The doctrine, that all fevers and all diseases, are essentially intermittent, has long been before the public; and while we are ignorant of the nature and source of periodicity, the fact of intermittence is as well established, as any other in medicine. And although a late writer has asserted boldly, and without exception, that *Therapeutics cannot be based on Pathology,* we aver as positively, that the direct opposite is true. We believe, especially in reference to fevers, almost without exception, that they are curable by sulphate of quinine, simply and solely, because it is a remedy, above all others, adapted to diseases of an intermittent and remittent character; their terms being substantially, and in fact, as to periodicity, the same.

In regard to the Pathology of Fevers, we know almost nothing, perhaps nothing at all, that is practically of the smallest value, save the naked fact of intermittence or periodicity.—This is cognisable when we can scarcely fix upon another point, that is worthy of notice or remark.

We cure an ague and fever, by sulphate of quinine, no matter whether it be a true and open ague, or a masked intermittent. And we cure many diseases of genuine neuralgia, unattended by chill or fever, at all perceptible, by the same agent. These are common cases, with which all practitioners are familiar, and they clearly set forth the adaptation of the remedy, on the ground of intermittence.

But if we look a little further, we shall discover, that other fevers, called by different names, merely to subserve the interests of theory, possess one common property, which, confessedly under the control of the sulphate of quinine in the case of common ague and fever, is not less so in Typhoid, Typhus, Congestive, Yellow, and it may be, all the fevers named in the books.

The position I assume here, is plainly and boldly this: *there is but one feature or element in either of the fevers named, that is essential to its pathology, and that feature or property or element bows before the potent sway of the sulphate of quinine, and for this reason only, we cure the patient.—Western Lancet.*

